# Year-Book of the Scientific and Learned Societies of Great Britain and Ireland

**Published:** 1891-06

**Authors:** 


*Year-Book of the Scientific and Learned Societies of Great Britain and Ireland.* Pp. 219. Eighth Annual Issue. London: CHARLES GRIFFIN AND COMPANY. 1891.

We gladly welcome the current volume of this admirable work of reference. As secretaries are not always careful to supply the information desired, any sins of omission which may be detected in it must be imputed to them and not to the compilers of the book. The lists of papers read are most useful.

